# A biological membrane-based novel excisional wound-splinting model in mice (With video)

**DOI:** 10.4103/2321-3868.143625

**Published:** 2014-10-25

**Authors:** Zhihui Yao, Yong Huang, Gaoxing Luo, Jun Wu, Weifeng He

**Affiliations:** State Key Laboratory of Trauma, Burns and Combined Injury, Institute of Burn Research, Southwest Hospital, The Third Military Medical University, Chongqing Key Laboratory for Proteomics of Diseases, Chongqing, 400038 China

**Keywords:** Animal model, biological membrane, re-epithelialization, wound healing

## Abstract

Rodents have robust wound healing mechanism compared to other animal species. The major mechanisms of wound healing differ between rodents and humans. In humans, wound healing primarily depends on re-epithelialization and granulation tissue (GT) formation, whereas wound contraction is more important during rodent wound closure. In this study, we described a novel excisional wound-splinting model in mice with a new biological membrane to imitate wound healing in humans. In this model, wound contraction can be effectually prevented, and the extent of re-epithelialization and the amount of granulation tissue can be determined easily. Furthermore, the harvested tissues can be analyzed with different methods according to the research aim. In conclusion, we have developed a biological membrane-based, novel, excisional wound-splinting model in mice that has unique advantages for wound healing research compared with the conventional animal model.

## Introduction

Wound healing is a dynamic and coordinated process, and there are various animal models used to study wound healing. Incontestably, rodents are used for a wide range of research applications due to low cost and ease of breeding and genetic modifications. However, the major mechanisms of wound healing differ between the humans and rodents. In humans, the skin closely connects to the subcutaneous tissues. Thus, wound healing depends on re-epithelialization and granulation tissue (GT) formation. In rodents, contraction is the major process of wound healing, because their skin is mobile and contains panniculus carnosus, which is a thin sheet of striated muscle between the subcutaneous fat and dermal layer.[1,2] Therefore, the rodent model does not precisely reflect human wound healing.

**Table 1:** 

Access this article online	
**Quick Response Code**:	**Website**: www.burnstrauma.com
	**DOI**: 10.4103/2321-3868.143625

In the present study, we describe a biological membrane-based, excisional wound-splinting model, which inhibits wound skin contraction efficiently to precisely reflect human wound healing.

## Materials and methods

In this study, we used C57BL/6 mice (males, 7–8 weeks old, 18–20 g) from the Animal Center of Daping Hospital in the Third Military Medical University. Animals were supplied with standard rodent diet and were housed in an animal facility (20–25°C and 12-hour light cycle).

## Procedure

### Animal preparation

Depilate the mice the day before surgery. Anesthetize the mice with an intraperitoneal injection of 1% sodium pentobarbital (10 µl/g).

Remove the fur from the dorsal skin (from neck to tail) with an electric clipper. Apply hair removal cream (Veet, 20070747) to the back for 3–5 min (not exceeding 6 min to avoid damaging the skin).

Clean the skin with the sterile cotton balls. Then, wipe again with sterile cotton balls soaked in warm water.

Keep the mice individually to avoid scratching and chewing.

Place the mice under a warm lamp until they are completely recovered.

### Skin wound procedure (With video)

Anesthetize the mice with an intraperitoneal injection of 1% sodium pentobarbital (10 µl/g).

Draw a line along the posterior midline, and draw another line along the root of the lower limbs (perpendicular to the first line). Mark points A and B on either side of the midline, 8 mm from the intersection.

Lift the dorsal skin slightly along the posterior midline. Perform 2 symmetrical 3 mm diameter, full-thickness, cutaneous biopsy punch wounds at point A and B. The wound sizes should be chosen according to your research aims and the strain of mice. Drugs, bioactive factors and cells can be applied to the wound bed or injected into the tissue to determine their effects on wound healing.

Glue the biological membrane (NPWT-1, Negative pressure wound therapy kit, China) with the adhesive dressings immediately onto the surface of the wound before contraction, and single-house the mice. For long-term wound observation, you should place simple, surgical sutures around the margin of the membrane to guarantee a higher success rate.

Place the mice under a warm lamp until they are completely recovered.

### Wound photographing

Anesthetize the mice with an intraperitoneal injection of 1% sodium pentobarbital (10 µl/g).

Place the contrast (the same diameter as the wound) beside the wound, and take photographs perpendicular to the individual wounds with a digital camera.

The photographing time points should be decided according to your research aims and should be discussed in the experimental design.

Place the mice under a warm lamp until they are completely recovered.

### Wound harvesting

Anesthetize the mice with an intraperitoneal injection of 1% sodium pentobarbital (10 µl/g).

Draw a circle along the 2 mm surrounding wound edge, and shear the entire wound tissue sample carefully along the circle. The wound tissue should be used according to your research purposes and should be discussed in the experimental design.

### Wound analysis

#### Wound size measurement

Measure the pixels of the actual wound and the control with Photoshop.

Calculate the percent wound closure as follows: (pixels of actual wound/pixels of control)/(pixels of original wound/pixels of control) × 100.

#### Histological analysis

The wound tissue should be harvested and divided across the center into two equal pieces. Carefully place the wound tissue on a piece of filter paper to avoid crispation. Fix the wound tissue in 4% paraformaldehyde at 4°C overnight.

The paraformaldehyde-fixed tissues can be embedded in paraffin, sectioned at 6 µm, and stained with hematoxylin and eosin (HE).

Examine the HE sections of the wound tissue with a light microscope. Then photograph the wound.

Wound re-epithelialization begins at the wound edges and moves toward the center. Take low-magnification images of the wound center and measure the extent of epithelialization and the gap between the epithelialized areas using Image-Pro Plus.

Measure the amount of GT at different time points. The HE sections are photographed under a light microscope, and the granulation thickness is calculated using Image-Pro Plus.

Specific markers can also be detected using immumohistochemical staining with specific antibodies according to your research purpose. Three to five animals are required in each time point.

### Fluorescence activated cell sorting (FACS) analysis

The harvested wound tissue can be incubated in 0.5% trypsin in 37° C for 30 min to digest single-cell suspensions.

Add an equal volume of phosphate-buffered saline (PBS) containing 3% fetal bovine serum (FBS) to the digest and strain through a 70 µm cell strainer.

Centrifuge the digest at 1000 rpm for 10 min at room temperature and resuspend the cells in 500 µl PBS with 3% FBS.

Count the cells with a cell counter and calculate the total cell number per wound.

Measure the percentage of green fluorescence protein (GFP)-positive cells or marked cells by FACS analysis in the single cell suspension.

### Anticipated results

**Figure 1:**
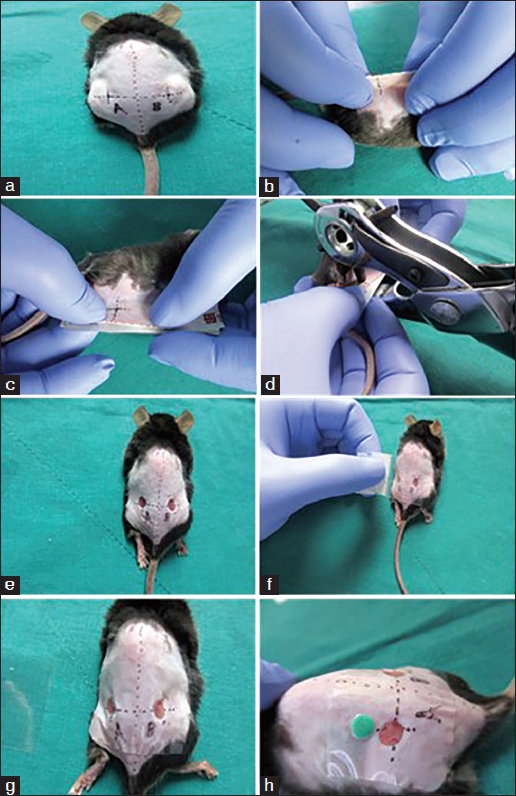
A biological membrane-based, novel, excisional, wound-splinting model in mice. (a) Draw point A and B to determine the position of the wound. (b and c) Lift the dorsal skin slightly along the posterior midline, and cushion the mouse with a paper pad. (d and e) Perform two full-thickness cutaneous biopsy punch wounds at points A and B. (f and g) Glue the biological membrane on the wound surface immediately before contraction. (h) Place the contrast beside the wound, and photograph the wound.

**Figure 2:**
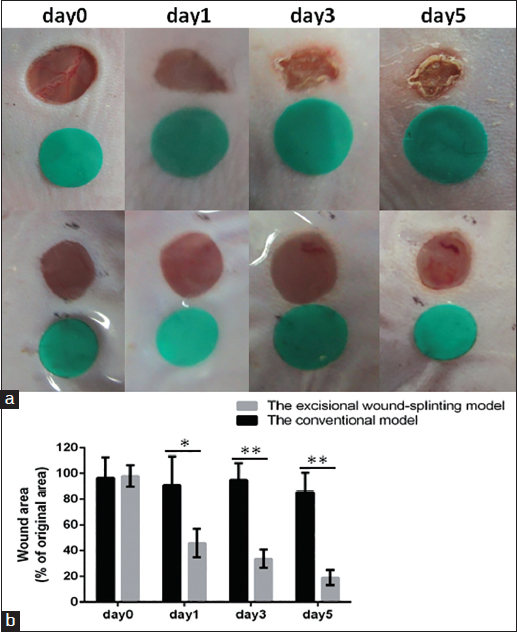
The biological membrane-based, novel, excisional, wound-splinting model minimizes wound closure caused by skin contraction compared with the conventional model in C57BL/6 mice. (a) The photographs of the excisional wound-splinting model and the conventional model at different time points. (b) The percentage of wound closure in the excisional wound-splinting model compared to the conventional model at each time point. Each point represents the mean wound area percentage of the original wound size ± standard deviation (SD) from 3–5 mice.

**Figure 3:**
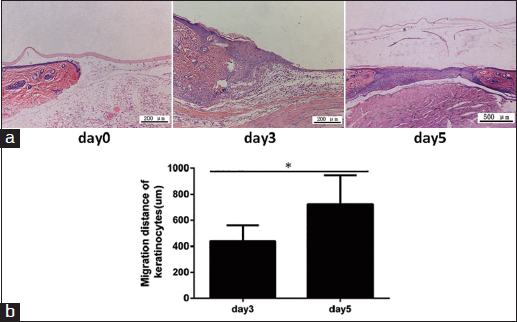
The re-epithelialization of the biological membrane-based, novel, excisional, wound-splinting model in C57BL/6 mice. (a) Histologic photographs on day 0, 3 and 5. (b) The extent of re-epithelialization is measured at different time points. Each point represents the mean migration distance of keratinocytes in the wound area ± standard deviation (SD) from 3–5 mice.

**Figure 4:**
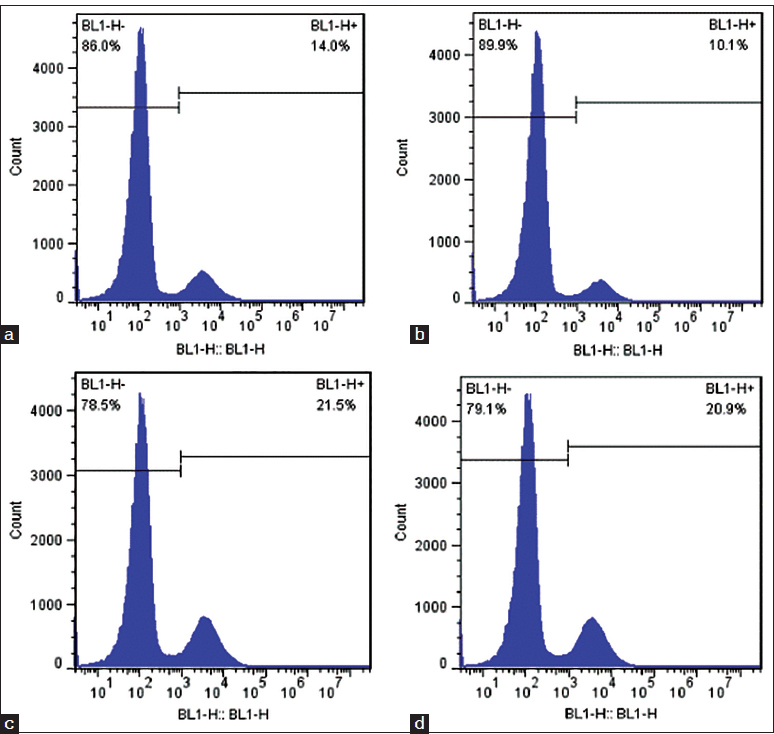
Fluorescence activated cell sorting analysis of single-cell suspensions from wound tissue to measure the percentage of green fluorescence protein (GFP)-positive cells in the biological membrane-based, novel, excisional, wound-splinting model compared with the conventional model in C57BL/6 mice. (a and b) The percentage of GFP-positive cells was 14.0% on day 3 and 10.1% on day 5 in the conventional model. (c and d) The percentage of GFP-positive cells was 21.5% on day 3 and 20.9% on day 5 in the biological membrane-based, novel, excisional, wound-splinting model.

Figure 1 demonstrates the steps of the biological membrane-based, novel, excisional and wound-splinting model [Figure 1]. This model minimizes wound closure caused by skin contraction compared to the conventional model. In the conventional model, wound closure will reach 30% in 48 h to the wound contraction. In this novel model, wound closure remained above 80% [Figure 2]. The extent of re-epithelialization should be measured using Image-Pro Plus at different time points [Figure 3]. The percentage of GFP-positive cells can be measured with FACS on day 3 and day 5 [Figure 4].

## Discussion

The major mechanisms of wound healing are different between rodents and humans due to differential skin organizational structure. Rodent models are not ideal because the major mechanism of wound healing in rodents is wound contraction, which is significantly different from that in humans.[2] In our model, we used a biological membrane, which is an adhesive dressing and is commonly used for continuous negative pressure therapy for the deep wounds. The biological membrane can glue the skin stably for 10 days. With the biological membrane, skin contraction can be efficiently prevented. The wound will heal by re-epithelialization and GT formation, which reasonably reflects human process. Additionally, this model is easy to perform and low cost. Drugs, bioactive factors and cells can be applied to the wound bed or injected into the tissue directly to determine their effects on wound healing and provide new treatment. Furthermore, with this model, all the genetic modifications and disease models in mice can be used to study wound healing. Meanwhile, the tissue harvested in this model can be used with various methods according the research aims (for example, with FACS analysis of single-cell suspensions or western blot analysis).

Some new animal wound models are also being used to prevent wound contraction in other studies. The rabbit ear dermal ulcer model is widely used as a splinted model. The rabbit ear skin is attached to the underlying cartilage, which restricts wound contraction and forces wound healing to occur via new tissue formation.[3] This model is used to investigate mechanisms of hypertrophic scaring and wound healing.[4] However, the disadvantage of this model is the high cost of breeding, and rabbits are hard to genetically modify. The cranial and tail excision models in rodents are other examples of anchored skin.[5,6] However, the underlying soft connective tissue is lacking, and these models require complex surgery. The excisional wound-splinting model described in the present study is a novel wound healing model in mice using wound-splinting, which efficiently prevents skin contraction and forces the wound to heal through granulation and re-epithelialization. Galiano *et al.* found that the rate of re-epithelialization in splinted wounds is similar to unsplinted wounds, and the amount of GT deposition is increased compared to unsplinted wounds.[7] Stem cells can be implanted into the wound bed to study cutaneous repair or regeneration using the mouse excisional, wound-splinting model.[8] In this model, special frames prevent wound contraction. For example, silicone rubber, metal rings and polydimethylsiloxane devices, require microfabrication techniques, and some laboratories cannot afford these techniques. These devices also require complex surgeries to fix the rings in the skin, which may aggravate the wound.[9,10] Chung *et al.* attached the Tegaderm HP dressing over the wound in diabetic and non-diabetic mice and found that the Tegaderm HP dressing retarded contraction in a large proportion of diabetic mice and, to a lesser extent, in non-diabetic mice.[11]

Here we describe in detail every operational step. For example, we confirmed the wound point according to the different lines drawn on the back, which is very important for the stability of this model and is not referenced in other articles. In our previous study, we found that the animal’s activities influence the position of the wound on the back. When the wound is on the top or middle of the back, the wound will be greatly influenced. In our study, the wound point placement is more constant and stable. Additionally, we propose more applications for this model. For example, drugs, bioactive factors and cells can be applied to the wound bed or injected into the tissue to determine their effects on wound healing, and the biological membrane could prevent the loss of drugs or cells.

In this study, we describe a new, excisional, wound-splinting model in mice to simulate human wound healing, which has some advantages compared to other animal models. We hope that this model can be used in more studies.
